# Temporal changes in cortical oxygenation in the motor-related areas and bilateral prefrontal cortex based on exercise intensity and respiratory metabolism during incremental exercise in male subjects: A near-Infrared spectroscopy study

**DOI:** 10.3389/fphys.2022.794473

**Published:** 2022-08-09

**Authors:** Sho Kojima, Shinichiro Morishita, Kazuki Hotta, Weixiang Qin, Naoto Usui, Atsuhiro Tsubaki

**Affiliations:** ^1^ Institute for Human Movement and Medical Sciences, Niigata University of Health and Welfare, Niigata, Japan; ^2^ Department of Physical Therapy, Kisen Hospital, Tokyo, Japan; ^3^ Department of Physical Therapy, Fukushima Medical University, Fukushima, Japan

**Keywords:** cortical oxygenation, near-infrared spectroscopy, incremental exercise, prefrontal cortex, supplementary motor area, primary motor cortex, anaerobic threshold, respiratory compensation point

## Abstract

A recent study has reported that prefrontal cortex (PFC) activity during incremental exercise may be related to exercise termination on exhaustion. However, few studies have focused on motor-related areas during incremental exercise. This study investigated changes in the oxygenation of the PFC and motor-related areas using near-infrared spectroscopy during incremental exercise. Moreover, we analyzed the effect of exercise termination on changes in cortical oxygenation based on exercise intensity and respiratory metabolism. Sixteen healthy young male patients participated in this study. After a 4-min rest and 4-min warm-up period, incremental exercise was started at an incremental load corresponding to 20 W/min. Oxyhemoglobin (O_2_Hb), deoxyhemoglobin (HHb), and total hemoglobin (THb) in the bilateral PFC, supplementary motor area, and primary motor cortex were measured. We evaluated changes in oxygenation in each cortex before and after the anaerobic threshold (AT) and respiratory compensation point to identify changes due to respiratory metabolism. O_2_Hb and THb increased from moderate intensity or after AT to maximal exercise, and HHb increased slowly compared to O_2_Hb and THb; these changes in hemoglobin levels were consistent in all cortical areas we measured. However, the increase in each hemoglobin level in the bilateral PFC during incremental exercise was faster than that in motor-related areas. Moreover, changes in cortical oxygenation in the right PFC were faster than those in the left PFC. These results suggest changes based on differences in neural activity due to the cortical area.

## 1 Introduction

Near-infrared spectroscopy (NIRS) can evaluate changes in cortical oxygenation during dynamic exercise such as running and cycling. Recently, changes in cortical oxygenation during submaximal exercise have received attention because brain activity is related to exercise termination on exhaustion (Robertson and Marino, 2016), but the underlying mechanisms are not clear. A previous study reported that oxyhemoglobin (O_2_Hb) in the prefrontal cortex (PFC) during incremental exercise reaches a peak at the respiratory compensation point (RCP) and decreases after RCP to maximal exercise, and deoxyhemoglobin (HHb) increases until maximal exercise (Rupp et al., 2008). In addition, the increase in O_2_Hb during incremental exercise for subjects with high exercise capacity is higher than that in subjects with low exercise capacity (Rupp et al., 2008; Oussaidene et al., 2015). The PFC has controlled cognitive and affective function (Ridderinkhof et al., 2004; Etkin et al., 2011), and motor-related areas such as the supplementary motor area (SMA), premotor area, and primary motor cortex (M1) have controlled motor output, coordination, and execution of movement (Nachev et al., 2008; Green et al., 2018; Côté et al., 2020). The PFC, SMA and M1 have indirect connectivity by premotor area (Robertson and Marino, 2016). The PFC is the region upstream of the motor-related area for motor control and determines exercise termination due to the integration of afferent feedback such as fatigue levels, physiological sensations and internal motivation levels (Robertson and Marino, 2016). The PFC may be activated earlier or higher than motor-related areas because aggregates multiple afferent feedback and forward to motor-related areas. Therefore, we have considered that activation in the PFC and motor-related areas during incremental exercise varied due to the different functions of these areas.

Several studies have reported different changes in cortical oxygenation between the PFC and motor-related areas during incremental exercise (Subudhi et al., 2009; Jung et al., 2015). Subudhi et al. (Subudhi et al., 2009) reported strong correlations between the left PFC, premotor, and motor regions with respect to HHb and total hemoglobin (THb), but not O_2_Hb or right PFC. Moreover, the increasing O_2_Hb in the PFC was greater in the right PFC than in the left. Therefore, right-PFC oxygenation during incremental exercise may change specifically, unlike in other areas. In addition, Jung et al. (Jung et al., 2015) reported that O_2_Hb in the PFC during incremental exercise significantly increased with rising exercise intensity, except in the motor cortex. Thus, cortical oxygenation in the PFC and motor-related areas may be different. However, previous studies have only compared exercise intensity with workload, and have not compared PFC and motor-related areas based on respiratory metabolism. Respiratory metabolism has two change points. The first point is the anaerobic threshold (AT), when as aerobic metabolism switches to anaerobic. The second point is the RCP, where the discharge of carbon dioxide is increased to buffer acidosis. Following AT point, arterial pH decreases until maximal exercise and more decreases from RCP (Wasserman et al., 1973). In addition, arterial partial pressure of carbon dioxide (PaCO_2_) decreases until maximal exercise after RCP (Wasserman et al., 1973; Smith and Ainslie, 2017). Decrease of arterial pH associate with vasodilatation, and increase cerebral blood flow due to vasodilatation (Kontos et al., 1977; Yoon et al., 2012). On the other hand, Decrease of PaCO_2_ related to decrease cerebral blood flow by the intermediary of vasoconstriction (Yoon et al., 2012; Smith and Ainslie, 2017). Cortical oxygenation may change based on the AT and RCP because they affect the circulation dynamics of the whole body. Therefore, measurement of changes in metabolism based on AT and RCP are important to understand changes in cortical oxygenation during incremental exercise. Breakpoints of cerebral oxygenation reported that both O_2_Hb and HHb increased concomitantly from the AT, and the HHb further increased while the O_2_Hb reached a plateau or decreased after the RCP (Racinais et al., 2014). We previously reported that O_2_Hb during incremental exercise only increases in the PFC, but not the premotor area, SMA or M1. However, the measurements our previous study were not differentiated by right or left PFC, and we included mostly female subjects (Kojima et al., 2020). The O_2_Hb, HHb and THb levels during exercise are reported higher male subjects compared with female subjects (Auger et al., 2016; Inagaki et al., 2021). Moreover, cerebral circulation in female is reportedly influenced by reproductive hormones (Barnes and Charkoudian, 2021). Therefore, sex difference is a factor to consider. The results of our previous study (Kojima et al., 2020) may include effects of sex difference; we need to exclude this effect. In addition, cerebral oxygenation reported to affect by changes of skin blood flow (SBF) (Miyazawa et al., 2013). Thus, measuring cortical oxygenation during incremental exercise need with consideration for effect of respiratory metabolism, sex, cortical areas and SBF. This study aimed to investigate changes in cortical oxygenation in the PFC and motor-related areas based on exercise intensity and respiratory metabolism during incremental exercise, and only include male subjects to consider sex difference. We hypothesized that cortical oxygenation of the right PFC at the AT changes independently from the left PFC and motor-related areas. This study may provide cortical oxygenation changes in multi-cortical areas during incremental exercise from a respiratory metabolism perspective. Our results may be an important for understanding the relationship between exercise and cortical oxygenation.

## 2 Materials and methods

### 2.1 Subjects

Sixteen healthy young male patients participated in this study. Participants had no fitness habits or history of neurological or orthopedic disorders and were unmedicated within the last 3 months. The study was approved by the Ethics Committee of Niigata University of Health and Welfare (approval number: 18082–181010) and conducted in accordance with the Declaration of Helsinki.

### 2.2 Experimental protocol

**FIGURE 1:**
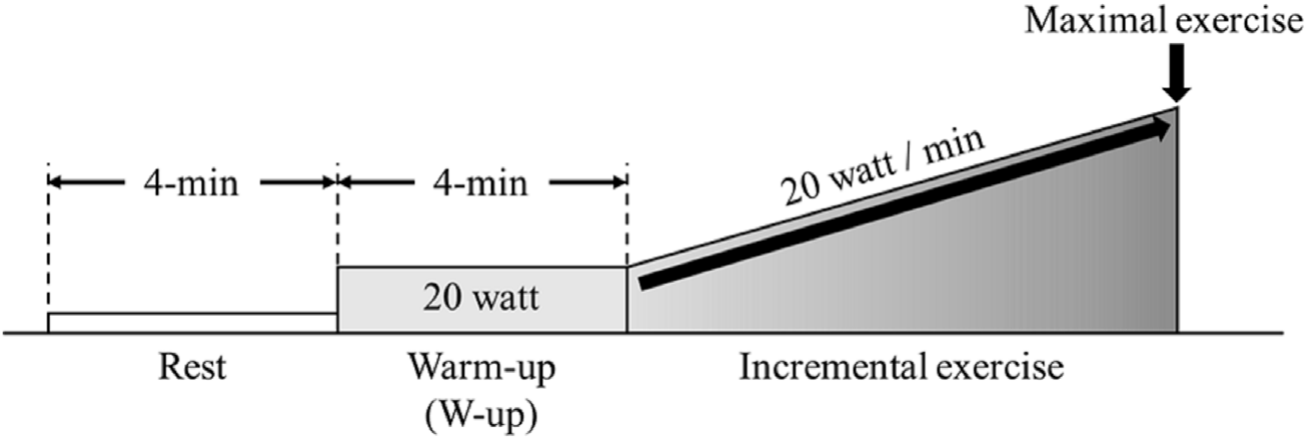
Experimental protocol. Participants performed incremental load exercise corresponding to 20 W/min after a 4-min resting state and warm up (W-up).

Participants were instructed not to consume alcohol or caffeine 24 h before the experiment. They were allowed to consume food and drink until 3 h before the experiments. Participants arrived 1 h prior to the start of experiment into the laboratory, wore experimental equipment, and had enough rest. Patients performed incremental exercise using the ramp load method on a cycle ergometer (Aerobike 75XLII; Combi, Tokyo, Japan) until exhaustion. Following a 4min rest, participants completed a 4 min warm up (W-up) pedaling at 20 W. Following the warm up the incremental protocol began increasing at a rate of 20W/min (Figure 1). The participants were instructed to maintain a cadence of 50–60 rpm. NIRS parameters, respiratory gas parameters, and skin blood flow (SBF) were measured from rest to the end of the incremental exercise.

### 2.3 Cortical oxygenation

**FIGURE 2:**
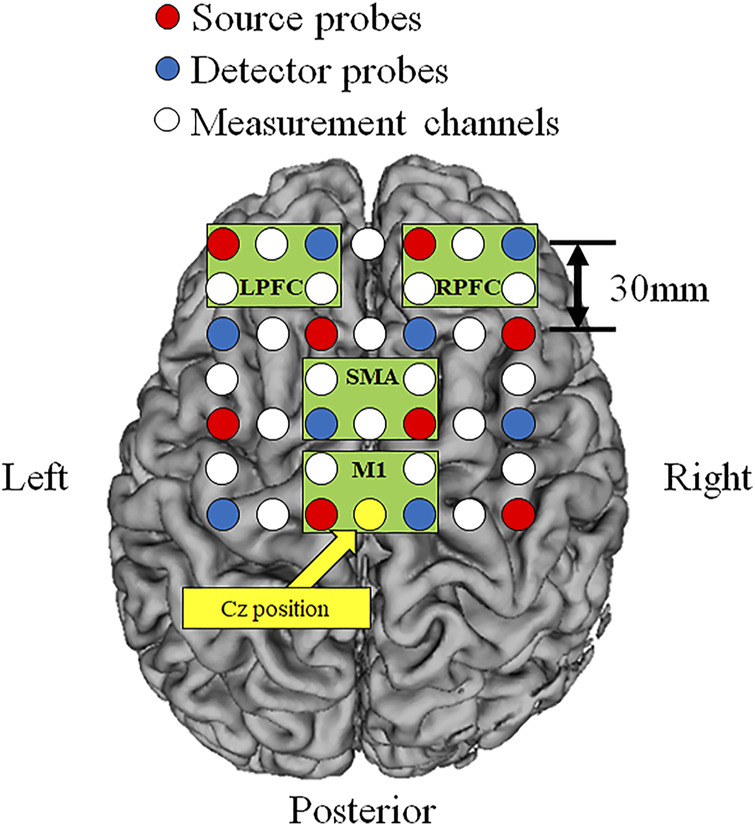
Locations of the source and detector probes and measurement channels. Red circles show source probes, blue circles show detector probes, white circles show measurement channels, and yellow circle show the vertex position (Cz). Green frames show measurement region of each cortex (L-PFC: left prefrontal cortex, R-PFC: right prefrontal cortex, SMA: supplementary motor area, M1: primary motor cortex).

Cortical O_2_Hb, HHb, and THb during incremental exercise were measured using a multi-channel NIRS imaging system (LABNIRS, Shimadzu Co., Kyoto, Japan) with multiple continuous wavelengths (780 nm, 805 nm, and 830 nm) based on the modified Beer-Lambert law. The regions of interest were the bilateral prefrontal cortex (left: L-PFC and right: R-PFC) and motor-related areas [including the SMA and primary motor cortex (M1)]. The measurement regions were standardized based on the vertex (Cz) position according to the international 10–20 system (Suzuki et al., 2008). The measurement channels included a total of 24 channels that used eight source probes and eight detector probes, and the probe distance was 30 mm (Figure 2). Measurement was performed at a sampling interval of 130 ms. Artifacts of head motion and heartbeat oscillations were filtered by a 0.1-Hz low pass filter (Qin et al., 2021). A repeat measurement was performed if obvious artifacts due to misalignment between head and NIRS system were confirmed.

### 2.4 Skin blood flow

We measured the SBF because a previous study reported the relationship between the SBF and cortical oxygenation (Miyazawa et al., 2013). The SBF during incremental exercise was measured with the midline of the forehead on 10 mm upward from the nasion of the international 10–20 system using a laser-tissue blood flow oxygen monitor (Omegaflow, FLO-Cl, Omega Wave Inc., Osaka, Japan). Analog data were converted to digital data using an A/D converter (PowerLab, AD Instruments, Australia) at a 130-Hz sampling rate.

### 2.5 Respiratory metabolism

Changes in oxygen uptake (VO_2_), carbon dioxide output (VCO_2_), minute ventilation (VE), partial pressure end-tidal oxygen (P_ET_O_2_), and partial pressure end-tidal carbon dioxide (P_ET_CO_2_) during incremental exercise were measured using a gas analyzer (AE-310, Minato Medical Science, Osaka, Japan). Respiratory metabolism data were obtained from breath-by-breath gas exchange data. We determined the AT and RCP based on previous studies (Wasserman and McIlroy, 1964; Wasserman et al., 1973). AT was determined by each point as follows: accelerating increase of VCO_2_ compared with VO_2_, increase of VE/VCO_2_ but not VE/VCO_2_, increase of respiratory exchange ratio, increase of P_ET_O_2_ but not P_ET_CO_2_. RCP determined by each point as follows: more increase of VE/VO_2_, P_ET_O_2_ and VE, increase of VE/VCO_2_, decrease of P_ET_CO_2_. Moreover, we calculated the peak value of VO_2_ (VO_2peak_) and VO_2_ at the AT and RCP and calculated the work rate (WR) and exercise time at the AT, RCP, and maximal exercise. Exhaustion was defined as follows: 1) a plateau in VO_2_; 2) respiratory exchange ratio >1.1; 3) heart rate values near the age-predicted maximal heart rate, calculated as 220—(0.65 × age); and 4) a decrease in the cycling cadence to <50 rpm, despite strong verbal encouragement (Morishita et al., 2018).

### 2.6 Statical analysis

Changes in O_2_Hb, HHb, THb, and SBF were calculated as the amount of change from the average of 4-min of rest. These parameters during rest and W-up were calculated from the average over 4-min. These parameters during incremental exercise were calculated from the average values of each 10th percentile (10%–100%) of the individual’s exercise time period from initiation to end exercise. In addition, these parameters were calculated as averages of 5-s intervals at timepoints where AT, RCP maximal exercise point (MAX), before 1-min of AT (before AT), and before 1-min of RCP (before RCP) occurred. Changes in O_2_Hb, HHb, THb, and SBF from rest to at W-up and during incremental exercise were compared using the one-way analysis of variance and the Bonferroni multiple comparisons test. Statistical analyses were performed using SPSS 21.0 (SPSS Japan Inc., Tokyo, Japan), with statistical significance set at *p* < 0.05.

## 3 Results

The patient age was 20.8 ± 0.4 years, BMI was 22.5 ± 2.7 kg/m^2^, VO_2_peak was 35.4 ± 5.4 ml/min/kg, WR at maximal exercise was 172.7 ± 34.3 W, exercise time at maximal exercise was 518.1 ± 102.9 s. The percentiles of maximal exercise time at the AT and RCP were 42.5 ± 5.7% and 86.3 ± 4.9%, respectively. VO_2_, exercise load, and exercise time at the AT and RCP are summarized in Table 1.

**TABLE 1:** Power output, oxygen uptake and exercise time during incremental exercise.

	Mean ± SD
Variable	
VO_2_ at AT (ml/min/kg)	17.4 ± 2.3
VO_2_ at RCP (ml/min/kg)	29.5 ± 4.0
VO_2_peak (ml/min/kg)	35.4 ± 5.4
%VO_2_peak at AT	49.6 ± 5.2
%VO_2_peak at RCP	83.8 ± 6.7
Exercise load at AT (watt)	74.0 ± 20.9
Exercise load at RCP (watt)	148.3 ± 27.2
Exercise load at maximal exercise (watt)	172.7 ± 34.3
time at AT (sec)	222.0 ± 62.8
time at RCP (sec)	444.9 ± 81.7
time at maxiaml exercise (sec)	518.1 ± 102.9
%maxmal exercise time at AT	42.5 ± 5.7
%maxmal exercise time at RCP	86.3 ± 4.9
VO_2_: oxygen uptake, VO_2peak_: peak oxygen uptake, AT: anerobic threshold, RCP: respiratory compensation point	

### 3.1 Changes in cortical oxygenation and SBF based on metabolism

The O_2_Hb significantly increased from AT in the L-PFC (AT: *p* = 0.04; before RCP, RCP and MAX: *p* < 0.001) and R-PFC (AT: *p* = 0.01; before RCP, RCP and MAX: *p* < 0.001) compared with rest. The O_2_Hb in the SMA and M1 showed significant increase from before RCP compared with rest (*p* < 0.001, respectively). The HHb significantly increased from before RCP in the R-PFC (*p* < 0.001, respectively) compared with rest. The HHb in the L-PFC and M1 showed significant increase from RCP compared with rest (L-PFC, RCP: *p* = 0.002, MAX: *p* < 0.001; M1, RCP: *p* = 0.01, MAX: *p* < 0.001), and the SMA showed significant increase of the HHb at MAX compared with rest (*p* = 0.006). The THb significantly increased from AT in the L-PFC (AT: *p* = 0.01; before RCP, RCP and MAX: *p* < 0.001) and R-PFC (AT: *p* = 0.003; before RCP, RCP and MAX: *p* < 0.001) compared with rest. The SMA and M1 showed significant increase of THb from before RCP compared with rest (*p* < 0.001, respectively). Moreover, the HHb significantly increased at MAX in the L-PFC (*p* = 0.03) and R-PFC (*p* < 0.001) compared with RCP. The SBF significantly increased before RCP compared with rest (Before RCP: *p* = 0.01; RCP and MAX: *p* < 0.001). These results are shown in Table 2 and Supplementary Table 1 and 2.

**TABLE 2:** Changes in cerebral oxygenation and skin blood flow based on respiratory metabolism.

Variable	Time						
	Rest	W-up	Before AT	AT	Before RCP	RCP	MAX
O_2_Hb × 10^–2^ (mM·cm)							
L-PFC	0.0 ± 0.0	0.7 ± 2.1	2.4 ± 3.3	4.2 ± 3.2*	7.7 ± 3.8*	8.7 ± 4.1*	7.3 ± 6.2*
R-PFC	0.0 ± 0.0	0.6 ± 1.6	2.2 ± 2.4	3.8 ± 2.5*	6.9 ± 2.7*	7.7 ± 3.4*	6.7 ± 5.2*
SMA	0.0 ± 0.0	0.2 ± 2.1	1.4 ± 2.3	3.0 ± 2.2	5.5 ± 2.6*	6.2 ± 3.7*	5.0 ± 5.6*
M1	0.0 ± 0.0	-0.1 ± 1.9	1.5 ± 2.4	2.6 ± 2.5	5.2 ± 2.7*	5.6 ± 3.1*	4.1 ± 3.5*
HHb × 10^–2^ (mM·cm)							
L-PFC	0.0 ± 0.0	0.0 ± 1.0	0.1 ± 1.6	0.6 ± 1.5	1.9 ± 2.3	3.2 ± 2.9*	5.7 ± 3.7*†
R-PFC	0.0 ± 0.0	0.3 ± 0.6	0.6 ± 1.0	0.9 ± 0.9	2.5 ± 1.4*	3.8 ± 1.8*	6.5 ± 2.5*†
SMA	0.0 ± 0.0	-0.1 ± 0.6	0.2 ± 1.1	0.4 ± 1.3	1.4 ± 1.6	2.2 ± 2.1	2.9 ± 4.7*
M1	0.0 ± 0.0	-0.8 ± 0.9	-0.6 ± 1.7	-0.2 ± 1.6	1.3 ± 1.8	2.4 ± 2.2*	4.5 ± 3.1*
THb × 10^–2^ (mM·cm)							
L-PFC	0.0 ± 0.0	0.7 ± 1.6	2.5 ± 2.9	4.8 ± 2.9*	9.7 ± 3.8*	11.9 ± 5.1*	13.1 ± 6.6*
R-PFC	0.0 ± 0.0	0.9 ± 1.5	2.8 ± 2.1	4.7 ± 2.4*	9.4 ± 3.1*	11.5 ± 4.0*	13.2 ± 5.9*
SMA	0.0 ± 0.0	0.2 ± 2.0	1.6 ± 1.9	3.4 ± 2.1	6.9 ± 2.9*	8.4 ± 4.3*	7.9 ± 9.2*
M1	0.0 ± 0.0	-0.9 ± 1.9	0.9 ± 2.6	2.4 ± 2.9	6.6 ± 3.4*	8.0 ± 3.6*	8.7 ± 4.8*
SBF (a.u.)	0.0 ± 0.1	-0.2 ± 0.6	0.8 ± 1.4	1.7 ± 2.1	4.6 ± 3.3*	7.5 ± 4.1*	8.8 ± 7.9*

O_2_Hb, oxyhemoglobin, HHb, deoxyhemoglobin, THb, total hemoglobin, SBF, skin blood flow, L-PFC, left prefrontal cortex, R-PFC, right prefrontal cortex, SMA, supplementary motor area, M1, primary motor cortex, W-up, warm-up, AT, anaerobic threshold, RCP, respiratory compensation point, MAX, maximal exercise point.

a *p*<0.05: significant different from the rest.

b *p*<0.05: significant different from the RCP.

Mean ± standard deviation.

### 3.2 Changes in cortical oxygenation and SBF at percentile

**FIGURE 3:**
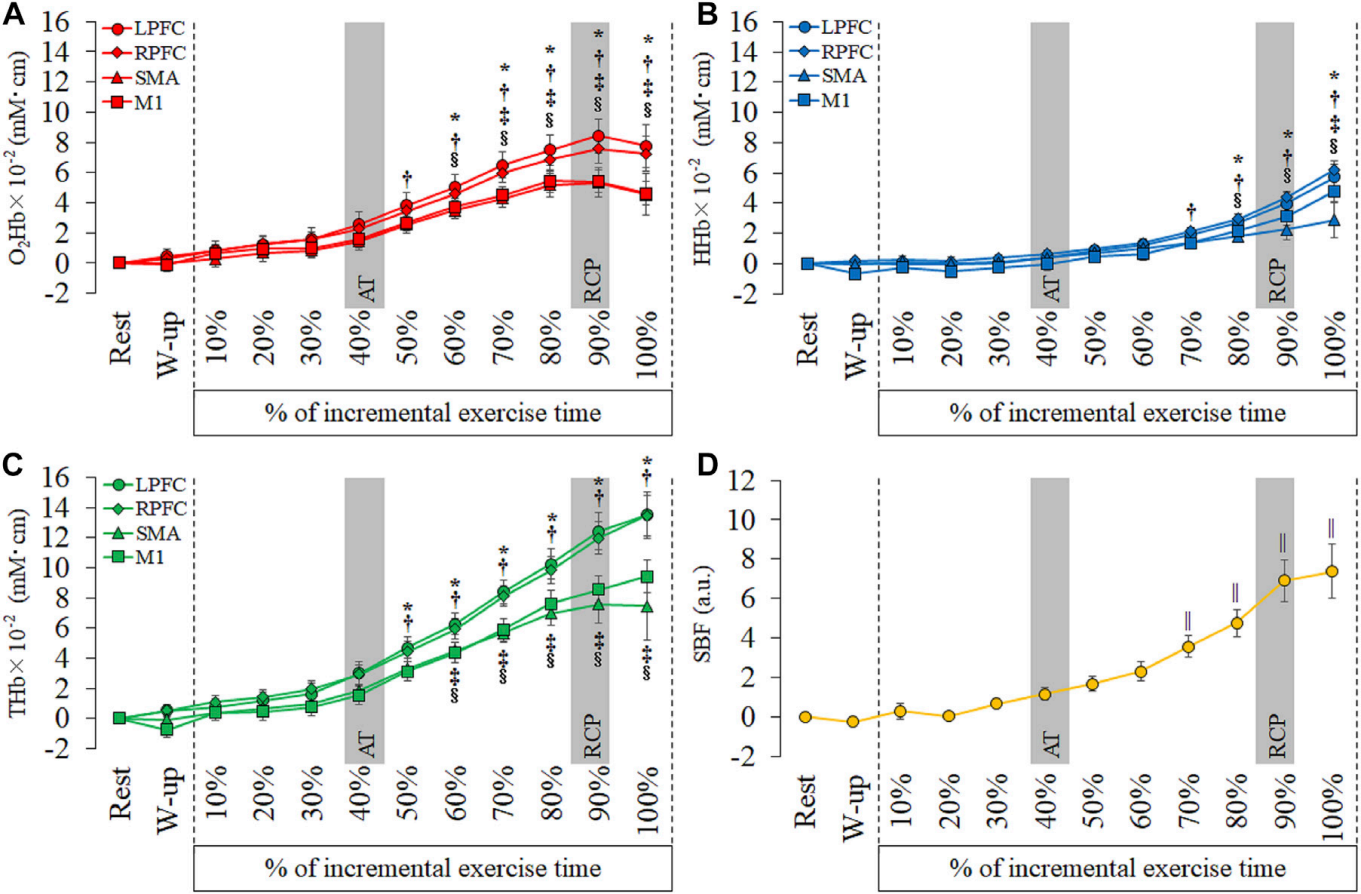
Temporal changes in each hemoglobin and skin blood flow (SBF) at each period of every 10^th^ of incremental exercise. **(A)**: oxyhemoglobin (O2Hb), **(B)**: deoxyhemoglobin (HHb), **(C)**: total hemoglobin (THb) and **(D)**: SBF. Region of interests are left prefrontal cortex (L-PFC), right prefrontal cortex (R-PFC), supplementary motor area (SMA) and primary motor cortex (M1). Gray bars show approximate position in the AT and RCP calculated from all participants. Significant different compared with rest: *p < 0.05.

As shown in Figure 3 and Supplementary Tables 3 and 4, the O_2_Hb significantly increased from 50% to 100% in the R-PFC compared with rest (50%: *p* = 0.02; 60%–100%: *p* < 0.001). The O_2_Hb in the L-PFC and M1 showed significant increase from 60% to 100% compared with rest (L-PFC, 60%: *p* = 0.005, 70%–100%: *p* < 0.001; M1, 60%: *p* = 0.001, 70%–100%: *p* < 0.001). The SMA showed significant increase of the O_2_Hb from 70 to 100% compared with rest (70%: *p* = 0.003, 80%–100%: *p* < 0.001). The HHb in the R-PFC significantly increased from 70% to 100% compared with rest (*p* < 0.001). The L-PFC and M1 showed significant increase of the HHb from 80% to 100% compared with rest (L-PFC, 80%: *p* = 0.005, 90% and 100%: *p* < 0.001; M1, 80%: *p* = 0.03, 90% and 100%: *p* < 0.001). The HHb in the SMA significantly increased from 100% compared with rest (*p* = 0.001). The THb significantly increased from 50% to 100% in the L-PFC (50%: *p* = 0.01, 60%–100%: *p* < 0.001) and R-PFC (50%: *p* = 0.002, 60%–100%: *p* < 0.001) compared with rest. The THb in the SMA and M1 significantly increased from 60% to 100% compared with rest (SMA, 60%: *p* = 0.04, 70%: *p* = 0.001, 80%–100%: *p* < 0.001; M1, 60%: *p* = 0.001, 70%–100%: *p* < 0.001). The SBF significantly increased from 70% to 100% compared to rest (70%: *p* = 0.006, 80%–100%: *p* < 0.001).

## 4 Discussion

We investigated changes in cerebral oxygenation in the bilateral PFC and motor-related areas based on changes in respiratory metabolism during incremental exercise. The main finding of our study was that the HHb in the R-PFC increased faster than in other cortical areas, and the HHb in the bilateral PFC further increased from RCP to MAX. In addition, the O_2_Hb and THb in the bilateral PFC during incremental exercise increased faster than those in the SMA and M1. This study is the first to reveal differences in cortical oxygenation changes between the PFC and motor-related areas based on respiratory metabolism.

### 4.1 Cerebral oxygenation during incremental exercise

In this study, we showed an increase in O_2_Hb, HHb, and THb with increasing intensity. These findings support previous findings regarding cerebral oxygenation during incremental exercise (Rupp et al., 2008; Giles et al., 2014; Kojima et al., 2020). Changes in cerebral oxygenation are reportedly affected by changes in SBF, cerebral blood flow (CBF), and cortical activity (Lindauer et al., 2010; Miyazawa et al., 2013). In general, NIRS signal at neural activity is related to an increase of O_2_Hb, decrease of HHb and an increase of THb, because the CBF increases with the cerebral metabolic rate of oxygen (CMRO_2_) (Lindauer et al., 2010). Increase in the HHb is caused by cortical activation, hypoxia, ischemia, or vein congestion (Murata et al., 2006; Subudhi et al., 2007; Lindauer et al., 2010). Subjective symptoms of hypoxia and ischemia were not observed in the subjects. Our study showed different timings of increase between SBF and cerebral oxygenation. The timepoint of significant increase in the O_2_Hb and THb were 50–60% during incremental exercise, while the SBF was 70% during incremental exercise (Figure 3). Moreover, previous studies reported none or negative correlation between the SBF and O_2_Hb at high intensity exercise and cool-down (Tsubaki et al., 2016; Kojima et al., 2021). Although SBF and O_2_Hb appear to increase simultaneously, we considered that changes in cerebral oxygenation were related to increased cerebral blood flow or cortical activation. CBF during incremental exercise increases with an increase in exercise intensity and reaches a plateau/decrease after high-intensity exercise (Smith and Ainslie, 2017). Changes in CBF and O_2_Hb during incremental exercise are similar and can be explained by arterial constriction/dilatation with P_ET_CO_2_ changes. However, a previous study reported that CBF increased with clamped P_ET_CO_2_ after RCP but did not increase O_2_Hb (Hansen et al., 2020). In addition, our previous study demonstrated no correlation between changes in cerebral oxygenation and P_ET_CO_2_ after RCP, and THb increased until maximal exercise (Kojima et al., 2021). Thus, changes in cerebral oxygenation during incremental exercise can be partially explained by CBF changes, while not explaining the changes from RCP to maximal exercise.

On the other hand, changes in cerebral oxygenation from the RCP to maximal exercise may be affected by neural activity in each cortex. Oxygen metabolism by neural activity accelerates conversion from the O_2_Hb to the HHb, whereas an increase in regional CBF exceeds the cerebral metabolic rate of oxygen (CMRO_2_) by a factor of 2–10, resulting in washout of HHb from the activation area, an increase in O_2_Hb, and a decrease in HHb (Lindauer et al., 2010). The PFC can aggregate afferent signals of affect, physical sensations, fatigue levels, and control to modify the exercise pace due to out via the pre-motor area and the basal ganglia (Robertson and Marino, 2016). Motor-related areas activation increases with the PFC because motor-related areas increase the power output of muscles with incremental exercise intensity (Brümmer et al., 2011). Cortical activation during incremental exercise is possibly increased from RCP to MAX owing to the maintenance of the exercise pace. However, neural activity in the PFC and motor cortex during incremental exercise, measured using electroencephalogram (EEG), was reported to decrease from RCP to maximal exercise (Robertson and Marino, 2015). In contrast, CMRO_2_ has been reported to increase until maximal exercise (Smith and Ainslie, 2017). Additionally, the systemic vascular conductance (VC) index and cerebral VC index increased with increasing load (Fisher et al., 2013), and systemic VC may increase the pulsatility index in the straight sinus, which shows resistance to venous outflow (Stolz et al., 2009). Imbalanced cerebral oxygenation from RCP to maximal exercise might be caused by a decrease in CBF, an increase in cerebral metabolic rate of oxygen, and a larger resistance to venous outflow, and these results may lead to a decline in neural activation.

### 4.2 Different of cerebral oxygenation in the prefrontal and motor-related areas

The O_2_Hb and THb in the bilateral PFC increased faster than in the SMA and M1, and the HHb in the R-PFC increased faster than in other cortical areas. A previous study that measured oxygenation in the PFC during incremental exercise reported that the non-linear changes in cerebral oxygenation were concomitant with both AT and RCP (Racinais et al., 2014). The O_2_Hb and THb levels in the PFC significantly increased from AT, and these results correspond with those of a previous study. On the other hand, the O_2_Hb and THb in the motor-related areas increased later compared to the bilateral PFC. This result shows that regional oxygenation changes during incremental exercise differ between PFC and motor-related areas.

Moreover, the HHb in the bilateral PFC increased MAX compared with the RCP. Increased HHb was not identified in motor-related areas. A previous study investigated the relationship between affective responses and cerebral oxygenation and reported a relationship between an increase in unpleasant emotion and an increase in the O_2_Hb at RCP and at the end of the exercise (Tempest et al., 2014). However, a previous study did not report a relationship between affective responses and HHb. Therefore, early increases in O_2_Hb and THb in the bilateral PFC and rapid increase in HHb from RCP to MAX may be affected by affective responses. Some studies have reported the extension of exercise time during incremental exercise by transcranial direct current stimulation (tDCS), which modulates cortical activity (Okano et al., 2015; Baldari et al., 2018). Anodal tDCS for the motor cortex extended the exercise time but did not change affective responses (Baldari et al., 2018). On the other hand, anodal tDCS for the insular cortex delayed the increase in the rating of perceived exertion and heart rate in addition to prolonged exercise time (Okano et al., 2015). The insular cortex has functional connectivity with the PFC and sends afferent feedback during exercise (Robertson and Marino, 2016; McMorris et al., 2018). The PFC is an important region that integrates afferent signals, such as fatigue, emotion, and perception of movement during exercise. Therefore, the oxygenation of the PFC may have increased earlier than in motor-related areas.

### 4.3 Limitations and strength

In this study, cortical activity, affective response, CMRO_2_, and CBF were not measured during the incremental exercise. Thus, differences in cortical oxygenation by function explicitly were not explained. Moreover, the amygdala and insular cortex related to affect are located deep in the brain. To understand cortical oxygenation and neural activity during incremental exercise, an overall assessment based on magnetic resonance imaging, positron-emission tomography, EEG, and NIRS is required.

We measured SBF using laser tissue blood flow oxygen monitoring. Measurement of the SBF is a strength of our research and removes the effect of extra-cerebral tissue. However, SBF was measured on the forehead. In particular, M1 is distant from the forehead, which is a limitation of this study. In addition, systemic physiological factors of cardiac output, mean arterial pressure and peripheral vascular resistance influence cerebral blood flow (Smith and Ainslie, 2017). We could not measure physiological factors other than skin blood flow, and this missing information is a limitation of our study. Future studies should assess SBF using short-channel regression methods and should measure physiological factors associated with cerebral blood flow and oxygenation.

However, few studies have measured NIRS during incremental exercise in multiple regions, and our study is the first to evaluate regional changes in cerebral oxygenation based on AT and RCP during incremental exercise. The present study may be an important study that increases the understanding of cortical oxygenation and neural activity during incremental exercise.

## 5 Conclusion

We found different changes in cerebral oxygenation in the PFC and motor-related areas during incremental exercise. The PFC during incremental exercise may be more active than the motor-related areas to continue exercise. Future studies are need to investigate the relationship between cerebral oxygenation in the multi-cortical area during incremental exercise, neural activity, fatigue, and emotion.

## Data Availability

The raw data supporting the conclusions of this article will be made available by the authors, without undue reservation.
