# Supplementary material for: Temporal changes in cortical oxygenation in the motor-related areas and bilateral prefrontal cortex based on exercise intensity and respiratory metabolism during incremental exercise in male subjects: A near-Infrared spectroscopy study
Source: Front Physiol. 2022 Aug 9;13:794473. doi: 10.3389/fphys.2022.794473 (PMC9396126; doi:10.3389/fphys.2022.794473)
Supplement: Supplementary file 1 [file DataSheet1.PDF]

Supplementary Material

1 Supplementary Figures and Tables

1.1 Supplementary Table 1.

Results of one-way ANOVA in changes in the cortical oxygenation and SBF based on respiratory metabolism		
**Variable**	**F-value**	**P-value**
O_2Hb		
L-PFC	F = 13.89	P < 0.001
R-PFC	F = 17.62	P < 0.001
SMA	F = 10.52	P < 0.001
M1	F = 12.89	P < 0.001
HHb		
L-PFC	F = 14.56	P < 0.001
R-PFC	F = 42.31	P < 0.001
SMA	F = 4.54	P < 0.001
M1	F = 16.57	P < 0.001
THb		
L-PFC	F = 29.40	P < 0.001
R-PFC	F = 39.79	P < 0.001
SMA	F = 11.24	P < 0.001
M1	F = 25.28	P < 0.001
SBF	F = 14.57	P < 0.001

ANOVA: analysis of variance, O_2Hb: oxyhemoglobin, HHb: deoxyhemoglobin, THb: total hemoglobin, SBF: skin blood flow, L-PFC: left prefrontal cortex, R-PFC: right prefrontal cortex, SMA: supplementary motor area, M1: primary motor cortex

1.2 Supplementary Table 2

Changes in cerebral oxygenation and skin blood flow based on respiratory metabolism.

	Time						
**Variable**	**Rest**	**W-up**	**Before AT**	**AT**	**Before RCP**	**RCP**	**MAX**
$O_2Hb\times10^{-2}$ (mM･cm)							
L-PFC	0.0±0.0	0.7±2.1	2.4±3.3	4.2±3.2	7.7±3.8	8.7±4.1	7.3±6.2
				* P = 0.04	* P < 0.001	* P < 0.001	* P < 0.001
R-PFC	0.0±0.0	0.6±1.6	2.2±2.4	3.8±2.5	6.9±2.7	7.7±3.4	6.7±5.2
				* P = 0.01	* P < 0.001	* P < 0.001	* P < 0.001
SMA	0.0±0.0	0.2±2.1	1.4±2.3	3.0±2.2	5.5±2.6	6.2±3.7	5.0±5.6
					* P < 0.001	* P < 0.001	* P < 0.001
M1	0.0±0.0	-0.1±1.9	1.5±2.4	2.6±2.5	5.2±2.7	5.6±3.1	4.1±3.5
					* P < 0.001	* P < 0.001	* P < 0.001
$HHb\times10^{-2}$ (mM･cm)							
L-PFC	0.0±0.0	0.0±1.0	0.1±1.6	0.6±1.5	1.9±2.3	3.2±2.9	5.7±3.7
						* P = 0.002	* P < 0.001
							† P = 0.03
R-PFC	0.0±0.0	0.3±0.6	0.6±1.0	0.9±0.9	2.5±1.4	3.8±1.8	6.5±2.5
					* P < 0.001	* P < 0.001	* P < 0.001
							† P < 0.001
SMA	0.0±0.0	-0.1±0.6	0.2±1.1	0.4±1.3	1.4±1.6	2.2±2.1	2.9±4.7
							* P = 0.006
M1	0.0±0.0	-0.8±0.9	-0.6±1.7	-0.2±1.6	1.3±1.8	2.4±2.2	4.5±3.1
						* P = 0.01	* P < 0.001
$THb\times10^{-2}$ (mM･cm)							
L-PFC	0.0±0.0	0.7±1.6	2.5±2.9	4.8±2.9	9.7±3.8	11.9±5.1	13.1±6.6
				* P = 0.01	* P < 0.001	* P < 0.001	* P < 0.001
R-PFC	0.0±0.0	0.9±1.5	2.8±2.1	4.7±2.4	9.4±3.1	11.5±4.0	13.2±5.9
				* P = 0.003	* P < 0.001	* P < 0.001	* P < 0.001
SMA	0.0±0.0	0.2±2.0	1.6±1.9	3.4±2.1	6.9±2.9	8.4±4.3	7.9±9.2
					* P < 0.001	* P < 0.001	* P < 0.001
M1	0.0±0.0	-0.9±1.9	0.9±2.6	2.4±2.9	6.6±3.4	8.0±3.6	8.7±4.8
					* P < 0.001	* P < 0.001	* P < 0.001
SBF (a.u.)	0.0±0.1	-0.2±0.6	0.8±1.4	1.7±2.1	4.6±3.3	7.5±4.1	8.8±7.9
					* P = 0.01	* P < 0.001	* P < 0.001

O_2Hb: oxyhemoglobin, HHb: deoxyhemoglobin, THb: total hemoglobin, SBF: skin blood flow, L-PFC: left prefrontal cortex, R-PFC: right prefrontal cortex, SMA: supplementary motor area, M1: primary motor cortex, W-up: warm-up, AT: anaerobic threshold, RCP: respiratory compensation point, MAX: maximal exercise point.
*: significant different from the rest. †: significant different from the RCP. Mean ± standard deviation.

1.3 Supplementary Table 3

Results of one-way ANOVA in changes in the cortical oxygenation and SBF at percentile		
**Variable**	**F-value**	**P-value**
O_2Hb		
L-PFC	F = 12.47	P < 0.001
R-PFC	F = 17.42	P < 0.001
SMA	F = 8.32	P < 0.001
M1	F = 12.29	P < 0.001
HHb		
L-PFC	F = 15.23	P < 0.001
R-PFC	F = 42.99	P < 0.001
SMA	F = 4.31	P < 0.001
M1	F = 15.16	P < 0.001
THb		
L-PFC	F = 31.75	P < 0.001
R-PFC	F = 39.78	P < 0.001
SMA	F = 10.93	P < 0.001
M1	F = 25.99	P < 0.001
SBF	F = 18.22	P < 0.001

ANOVA: analysis of variance, O_2Hb: oxyhemoglobin, HHb: deoxyhemoglobin, THb: total hemoglobin, SBF: skin blood flow, L-PFC: left prefrontal cortex, R-PFC: right prefrontal cortex, SMA: supplementary motor area, M1: primary motor cortex

1.4 Supplementary Table 4

	Changes in cerebral oxygenation and skin blood flow at percentile.											
	Time											
**Variable**	**Rest**	**W-up**	**10%**	**20%**	**30%**	**40%**	**50%**	**60%**	**70%**	**80%**	**90%**	**100%**
$O_2Hb \times 10^{-2}$ (mM・cm)												
L-PFC	0.0±0.0	0.5±1.9	0.8±2.5	1.3±2.4	1.6±3.1	2.6±3.4	3.8±3.3	5.0±3.4	6.5±3.5	7.5±4.0	8.4±4.6	7.8±5.7
								P = 0.005	P < 0.001	P < 0.001	P < 0.001	P < 0.001
R-PFC	0.0±0.0	0.3±1.3	0.8±1.7	1.3±1.8	1.6±2.1	2.3±2.5	3.5±2.7	4.6±2.7	6±2.5	6.9±3.0	7.6±3.8	7.3±4.6
							P = 0.02	P < 0.001	P < 0.001	P < 0.001	P < 0.001	P < 0.001
SMA	0.0±0.0	0.0±1.9	0.3±2.2	0.7±2.2	0.8±2.1	1.4±2.3	2.5±2.2	3.5±2.3	4.3±2.3	5.2±3.1	5.3±3.9	4.6±5.6
									P = 0.003	P < 0.001	P < 0.001	P = 0.001
M1	0.0±0.0	-0.1±1.9	0.6±2.3	0.9±2.1	1.0±2.2	1.6±2.2	2.7±2.3	3.7±2.2	4.5±2.2	5.4±3.0	5.4±3.0	4.6±3.2
								P = 0.001	P < 0.001	P < 0.001	P < 0.001	P < 0.001
$HHb \times 10^{-2}$ (mM・cm)												
L-PFC	0.0±0.0	0.1±1.0	-0.1±1.4	0.0±1.3	0.1±1.5	0.4±1.4	0.9±1.5	1.3±1.6	1.9±1.8	2.7±2.3	4.0±2.8	5.7±3.5
										P = 0.005	P < 0.001	P < 0.001
R-PFC	0.0±0.0	0.2±0.7	0.2±1.0	0.2±1.0	0.4±0.9	0.7±0.9	1.0±0.9	1.4±0.9	2.1±1.0	3.0±1.3	4.4±1.7	6.2±2.2
									P < 0.001	P < 0.001	P < 0.001	P < 0.001
SMA	0.0±0.0	0.0±0.6	0.1±0.8	0.0±0.9	0.1±1.1	0.4±1.1	0.7±1.1	1.0±1.4	1.4±1.5	1.8±1.7	2.2±2.6	2.9±4.7
												P = 0.001
M1	0.0±0.0	-0.6±0.9	-0.3±1.2	-0.5±1.2	-0.2±1.5	0.0±1.6	0.4±1.5	0.6±1.5	1.4±1.7	2.2±1.7	3.1±2.3	4.8±3.0
										P = 0.03	P < 0.001	P < 0.001
$THb \times 10^{-2}$ (mM・cm)												
L-PFC	0.0±0.0	0.5±1.6	0.8±2.0	1.2±2.2	1.6±2.9	3.0±3.1	4.7±2.8	6.3±2.9	8.4±3.1	10.3±4	12.4±4.9	13.5±6.2
							P = 0.01	P < 0.001	P < 0.001	P < 0.001	P < 0.001	P < 0.001
R-PFC	0.0±0.0	0.5±1.2	1.1±1.6	1.5±1.7	2.0±2.1	2.9±2.5	4.4±2.7	5.9±2.7	8.1±2.8	9.8±3.5	12.0±4.3	13.5±5.4
							P = 0.002	P < 0.001	P < 0.001	P < 0.001	P < 0.001	P < 0.001
SMA	0.0±0.0	-0.1±1.8	0.4±1.9	0.7±1.9	0.9±1.8	1.8±1.9	3.3±1.8	4.5±2.2	5.7±2.4	7.0±3.3	7.6±5.1	7.5±9.2
								P = 0.04	P = 0.001	P < 0.001	P < 0.001	P < 0.001
M1	0.0±0.0	-0.8±2	0.4±2.0	0.5±2.2	0.7±2.4	1.5±2.4	3.1±2.6	4.4±2.6	5.9±2.9	7.6±3.5	8.5±3.6	9.4±4.5
								P = 0.001	P < 0.001	P < 0.001	P < 0.001	P < 0.001
SBF (a.u.)	0.0±0.0	-0.2±0.6	0.3±1.6	0.0±0.9	0.7±0.9	1.2±1.3	1.7±1.4	2.3±1.9	3.6±2.2	4.8±2.7	6.9±4.2	7.4±5.4
									P= 0.006	P < 0.001	P < 0.001	P < 0.001

O_2Hb: oxyhemoglobin, HHb: deoxyhemoglobin, THb: total hemoglobin, SBF: skin blood flow, L-PFC: left prefrontal cortex, R-PFC: right prefrontal cortex, SMA: supplementary motor area, M1: primary motor cortex, W-up: warm-up

P-values showed comparison between rest and each percentile. Mean ± standard deviation.
